# Plasma-assisted multiscale topographic scaffolds for soft and hard tissue regeneration

**DOI:** 10.1038/s41536-021-00162-y

**Published:** 2021-09-09

**Authors:** Woochan Kim, Yonghyun Gwon, Yang-Kyung Kim, Sunho Park, Sung-Ju Kang, Hyeng-Kyu Park, Myung-Sun Kim, Jangho Kim

**Affiliations:** 1 Department of Rural and Biosystems Engineering, Chonnam National University, Gwangju, Republic of Korea; 2 Interdisciplinary Program in IT-Bio Convergence System, Chonnam National University, Gwangju, Republic of Korea; 3 Department of Orthopedics, Chonnam National University Hospital, Gwangju, Republic of Korea; 4 Department of Physical and Rehabilitation Medicine, Chonnam National University Medical School & Hospital, Gwangju, Republic of Korea; 5 Institute of Nano-Stem Cells Therapeutics, NANOBIOSYSTEM Co., Ltd, Gwangju, 61008 Republic of Korea

**Keywords:** Tissues, Regenerative medicine, Tissue engineering

## Abstract

The design of transplantable scaffolds for tissue regeneration requires gaining precise control of topographical properties. Here, we propose a methodology to fabricate hierarchical multiscale scaffolds with controlled hydrophilic and hydrophobic properties by employing capillary force lithography in combination with plasma modification. Using our method, we fabricated biodegradable biomaterial (i.e., polycaprolactone (PCL))-based nitrogen gas (N-FN) and oxygen gas plasma-assisted flexible multiscale nanotopographic (O-FMN) patches with natural extracellular matrix-like hierarchical structures along with flexible and controlled hydrophilic properties. In response to multiscale nanotopographic and chemically modified surface cues, the proliferation and osteogenic mineralization of cells were significantly promoted. Furthermore, the O-FMN patch enhanced regeneration of the mineralized fibrocartilage tissue of the tendon–bone interface and the calvarial bone tissue in vivo in rat models. Overall, the PCL-based O-FMN patches could accelerate soft- and hard-tissue regeneration. Thus, our proposed methodology was confirmed as an efficient approach for the design and manipulation of scaffolds having a multiscale topography with controlled hydrophilic property.

## Introduction

Designing functional scaffolds to effectively replace, repair, or engineer human tissues and organs is one of the most powerful strategies in the fields of regenerative medicine and tissue engineering^1,2^. The mechanical properties and architectures of scaffolds serving as transplantable platforms should be precisely controlled for their application to various tissues and organs^3,4^. In particular, transplantable scaffolds should possess appropriately flexible and foldable characteristics consistent with the anatomical site of the implant^5^. Such flexible and foldable properties allow for the scaffolds to attach well to various structures such as the irregular surfaces of biological tissues or organs and facilitate handling during surgical implantation. In addition, these properties are important for the treatment of tissues (e.g., bone, cartilage, muscle, tendon, ligament, and skin) that must be guaranteed for motility during implantation. However, the design of a functional scaffold requires considerations beyond mechanical properties. In particular, the architecture and structure of scaffolds must be considered alongside their mechanical properties to guarantee stable support as well as ensure an active nutrient supply to the regeneration site, cell adhesion induction, and biological interactions toward cells or tissues^6^. Therefore, precisely designing scaffolds based on the biophysical properties of tissues and organs is a powerful approach for regenerative medicine.

In vivo, living cells are exposed to and surrounded by cellular microenvironments comprised of chemical factors, mechanical factors, cell–cell interactions, and structural factors of the extracellular matrix (ECM), which can eventually be converted into biological signals to regulate the functions, fates, and behaviors of cells that ultimately influence tissue formation and function^7–12^. The well-defined organization of the ECM with complex micro- and nanoscale topographic features suggests its crucial role in the regulation of physiologically relevant cellular functions (i.e., morphology, migration, proliferation, and differentiation) in vivo^7,13^. Accordingly, obtaining a structural understanding of the multiscale topography of the ECM can provide important insights for the efficient design and development of scaffolds for cell and tissue engineering. Several recent studies have demonstrated that scaffolds designed according to the multiscale topographical organization of ECMs showed improvement in behaviors and functions of cells along with enhanced tissue regeneration^14–16^. Importantly, scaffolds designed by mimicking highly aligned micro- and nanotopographical features of the bone ECM were found to promote not only the fate and functions of stem cells but also induce bone tissue regeneration^17,18^. Accordingly, the application of such scaffolds that can effectively mimic the multiscaled environment of the well-organized and highly aligned ECM has been highlighted as a promising strategy for accelerating tissue regeneration by improving cell function.

Numerous technologies and approaches have been applied to develop scaffolds that mimic the topographical characteristic of the ECM for clinical use, such as three-dimensional printing, electrospinning, and lithography^19,20,21^. In particular, such technologies are greatly beneficial to recreate the micro- or nanostructures and topography owing to their properties of high resolution, size controllability, and large-area processing without requiring sophisticated equipment and complex processes^22–24^. Although various scaffolds with a simple micro- or nanotopography have been achieved, developing a complex multiscale scaffold with a uniformly multiplex-nanosized topography or different nanostructures remains a challenge owing to the need for sophisticated processing steps and limitations of appropriate fabrication techniques. Even when such complex designs can be constructed, developing the large-scale multiplex-nanosized scaffolds that can be applied to various tissues or organs for a variety of purposes and covers defects, injuries, wound, or repairable site of several tens of centimeters of organs or tissues is limited by current technical difficulties. Above all, it is crucial to design and manipulate scaffolds with controlled multiscale surface properties for cell and tissue engineering^25,26^.

Guided by these considerations, we here propose a new approach for scaffold design involving the manipulation of a plasma-assisted flexible multiscale nanotopographic patch, which has multiscale structures with an aligned nanotopography and etched nanoporous topography, by employing capillary force lithography (CFL) in combination with plasma treatment to mimic the porous structure of the ECM. For this purpose, we fabricated US Food and Drug Administration (FDA)-approved polycaprolactone (PCL)-based N_2_ gas- (N-FN) or O_2_ gas plasma-assisted multiscale nanotopographic (O-FMN) patches that showed natural ECM-like hierarchical structures, including highly aligned nanoscale matrix (ridges and grooves of ~800 nm) with nanosized pores (~100 nm), along with flexible and controlled hydrophilic properties. Using these O-FMN patches as scaffolds, we investigated the influence of the multiscale hierarchical topography and chemically modified surface cues on the proliferation and osteogenic mineralization of cells. In addition, as a proof-of-concept, we investigated the influence of the O-FMN patches on the regeneration of the rotator cuff tendon tissue and calvarial bone tissue in the animal models. Furthermore, we quantitatively investigated the relative contributions of aligned nanotopographic cues, nanoporous structure cues, and chemically modified surface cues on cellular behaviors and tissue regeneration.

## Results

### Characteristics and properties of N-FN patches

The abbreviations for all samples in this work are as follows: flexible flat patch (FF patch), flexible nanotopographic patch (FN patch), N_2_ gas plasma-treated flexible flat patch (N-FF patch), N_2_ gas plasma-treated flexible nanotopographic patch (N-FN patch), O_2_ gas plasma-treated flexible flat patch (O-FF patch), O_2_ gas plasma-treated flexible multiscale nanotopographic patch (O-FMN patch).

**Fig. 1:**
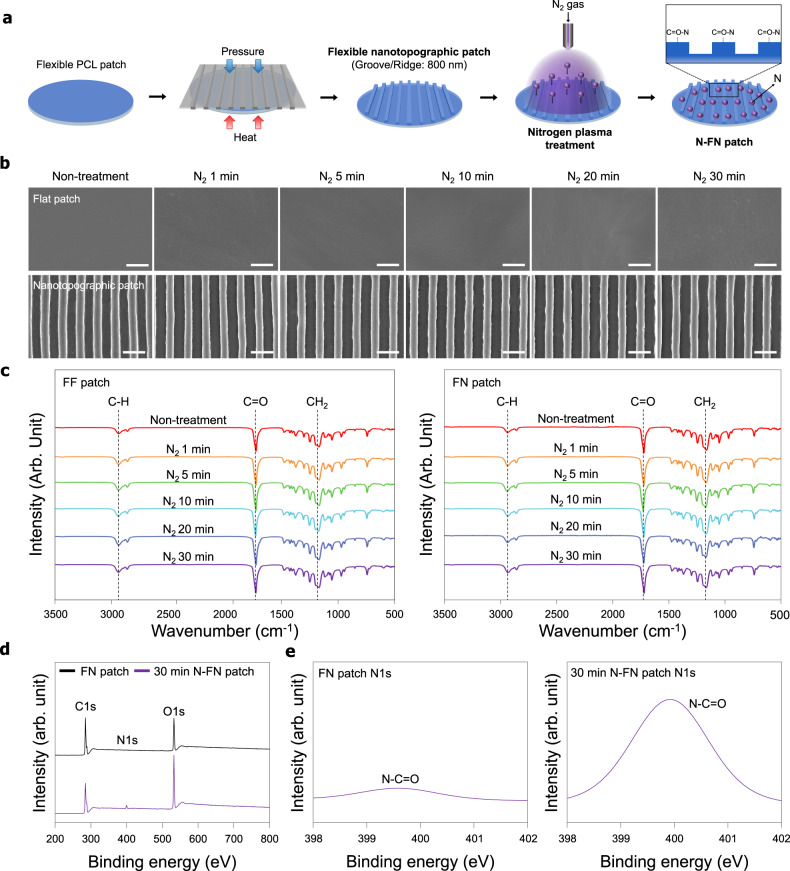
Schematic of the fabrication and characterization of a N-FN patch. **a** Fabrication of a polycaprolactone (PCL)-based FN patch. Inspired by the high aligned and well-organized nanotopography of the native ECM, the PCL-based FN patch was fabricated using capillary force lithography (CFL). Plasma surface modification on the surface of the FN patch. The surface of the patches was modified using N_2_ plasma reaction gas. **b** SEM images of the surface of FN patches treated by N_2_ plasma reaction gas under various times. Scale bars = 2 µm. **c** FT-IR analysis of the N-FF and N-FN patches treated plasma at various times. **d** XPS survey scans and **e** high-resolution N1s XPS spectra of FN patches and 30-min N-FN patches.

Figure 1a shows a schematic of the plasma treatment process used in this study (described in detail in “Methods”). In our previous works, we reported the structures and topography on microenvironment of natural tendon and bone ECMs^17,27^. Briefly, native tendons consist of well-organized and highly aligned collagen fibers in ECMs with ~86% type I collagen of type I and small amounts of type III collagen. These collagen fibers were cross-linked with proteoglycans, revealing closely packed parallel structures^28^. In the tendon ECM, a collagen fiber is composed of a large number of fibrils. Collagen fibers that come together to form collagen fibers vary in diameter from 500 nm to 1 μm. Collagen fibers are assembled to form bundles (or fascia) that are ~10 mm long and 1–20 μm in diameter. These fiber bundles finally assemble to form tendon units 20–500 μm in diameter^29^. Cells are mainly located between the collagen fibers and are affected by the aligned collagen parallel array structure and pores which contributed to the exchange of oxygen and provided nutrition. Furthermore, the aligned nanotopographies of collagen fibers and nanopores are similarly observed in bone extracellular matrix^17^. SEM images of the surface morphology of N-FN patches revealed a highly aligned topography with grooves and ridges (~800 nm size), similar to the well-organized topography of the native tendon ECM, without deformation and etching of surfaces with increasing plasma treatment times (Fig. 1b).

**Fig. 2:**
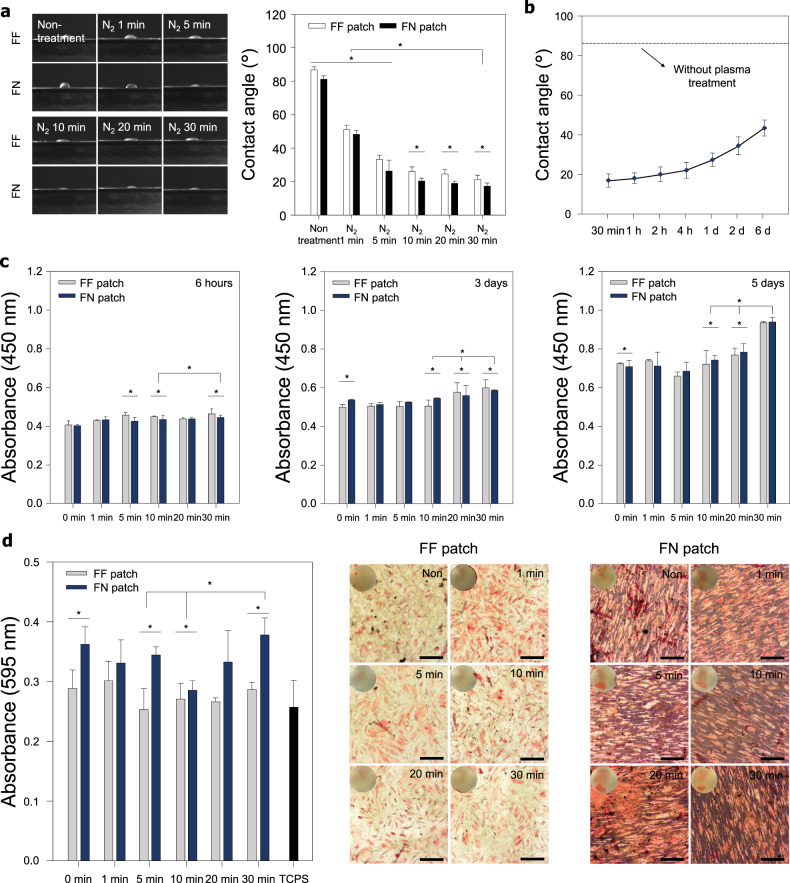
Effect of N-FN patches on cellular behaviors. **a** Water contact angle measurement of the N-FN treated under various times (*n* = 10 for each group). **b** Wettability recovery on the surfaces of 30-min N-FN patches (*n* = 5). **c** Attachment and proliferation of cells on N-FN patches. Quantitative analysis of cell attachment and proliferation on N-FF patches and N-FN patches showed a gradual increase with plasma treatment time (*n* = 6 for each group). **d** Effect of N-FN patches on the osteogenic mineralization of tenocytes. Alizarin Red staining and quantification of the degree of osteogenesis showed that the 30-min N-FN patch promoted higher calcium expression levels of tenocytes when compared with other groups (*n* = 6 for each group). Scale bars = 200 µm. Error bars = mean ± standard deviation (*P < 0.05).

To verify whether the N_2_ reaction gas and various plasma treatment time conditions used in the plasma treatment process affected PCL properties, the polymer’s chemical characteristics were analyzed. The functional groups of plasma-treated patches were investigated by FT-IR spectroscopy (Fig. 1c). The characteristic absorption bands related to PCL (i.e., CH_2_ asymmetric stretching at 2944 cm^−1^, symmetric stretching at 2866 cm^−1^, C = O stretching vibration of carbonyl groups at 1721 cm^–1^, and deformation of C–O at 1161 cm^–1^) were detected in all patches. The chemical changes of the plasma-treated PCL patches were not detected compared to the PCL patches. The surface chemical composition of the N-FN patches was analyzed by XPS. Comparison of the survey scan spectra of FN and N-FN patches showed three separated peaks in all XPS spectra, which correspond to C1s (285 eV), N1s (400 eV), and O1s (532 eV) (Fig. 1d). A distinct N1s peak at 400 eV in the N-FN patch spectrum indicated that N_2_ plasma was successfully applied onto the FN patch. The surface atomic compositions of the FN patch were calculated to be 73.91%, 0.29%, and 25.81% for C1s, N1s, and O1s, respectively. The surface atomic compositions of the N-FN patch were calculated to be 61.83%, 3.34%, and 34.82% for C1s, N1s, and O1s, respectively. The high-resolution XPS N1s spectra of the N-FN patches showed that the N1s peaks of the FN and N-FN patches can be decomposed into a component: one main N–C = O (399.9 eV) (Fig. 1e). The atomic configuration of the FN patch was calculated to be 0.51% and those of N-FN was 2.96% for N–C = O. Assessment of wettability of patches through measurement of the water contact angle showed that FN patches have a lower contact angle (82.56 ± 1.8°) when compared with FF patches (88.32 ± 1.7°) (Fig. 2a). The water contact angle of both FF and FN patches gradually decreased with increasing N_2_ plasma treatment time, and the 30-min N-FN patches had a lower contact angle (17 ± 1.4°) than that of 30-min N-FF patches (21 ± 2.1°). The wettability and hydrophilicity of polymer surface treated with plasma are transient. The hydrophobic properties of synthetic polymers are restored several hours after plasma treatment because uncured hydrophobic polymer chains migrate to the surface^30^. These phenomena, in addition to the oxidation process, include charge leakage from the surface, as well as migration and diffusion and redirection of polar groups, and therefore depend on the surface treatment conditions, material properties, and storage conditions^31^. Accordingly, we conducted the hydrophobic recovery analysis to confirm the maintenance of hydrophilicity and the storage period of scaffold surfaces in air and room temperature conditions immediately after the plasma treatment. The plasma-treated scaffolds were used immediately after the plasma treatment for in vitro and in vivo experiments within an hour. To confirm the hydrophobicity variation on the surface after N_2_ plasma treatment on the FN patch, static contact angle measurements were made at various time points (30 min, 1 h, 2 h, 4 h, 1 day, 2 days, and 6 days) after plasma treatment (Fig. 2b). N-FN patches exhibited slight hydrophobic recovery at 30 min to 4 h and showed substantial hydrophobic recovery from 1 to 6 days after plasma treatment. However, the hydrophilicity of N-FN patches was maintained when compared with the static water contact angle of FN patches.

### In vitro analysis of cell behavior on N-FN patches

To investigate whether the N_2_ plasma treatment on the surfaces of FF and FN patches influenced cell proliferation and attachment, we cultured human tenocytes on the patches for 6 h (cell attachment assay), 3 days (cell proliferation assay), and 5 days (cell proliferation assay), respectively (Fig. 2c). After 6 h of cell culture, unattached cells were removed by washing with PBS, and cells attached to the patches were quantified by the WST-1 assay. Tenocytes were well attached on all patches, irrespective of topographic properties, and cells on the 30-min N-FF and N-FN patches showed higher attachment than did those under other plasma treatment times. After 3 and 5 days of cell culture, cell proliferation was higher on the 30-min plasma treatment patches when compared with those of the other groups (Fig. 2c). The osteogenic mineralization of tenocytes on the N-FN patches was examined by culturing cells on the two scaffolds in osteogenic induction medium for 14 days. Alizarin Red staining (Fig. 2d) revealed slightly higher calcium expression levels on the 30-min N-FN patches than on other samples and the tissue culture polystyrene substrate (TCPS). Although the N-FN patches treated for 30 min showed the highest degree of quantification on the osteogenic mineralization, there was no significant difference from the other control groups.

### Characteristics and properties of O-FMN patches

**Fig. 3:**
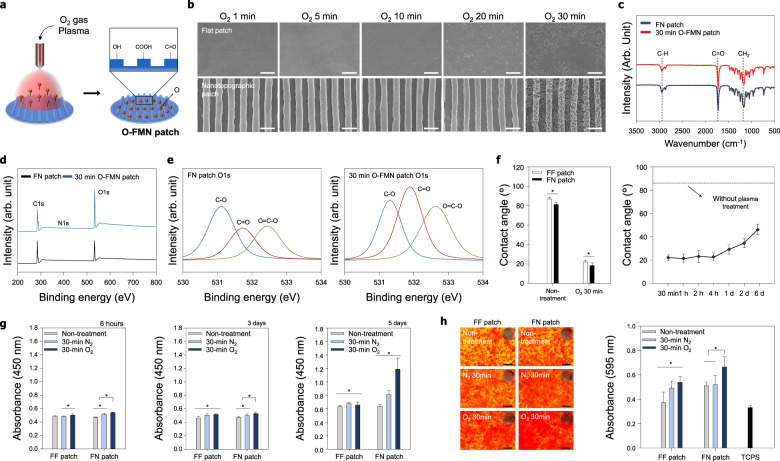
Characterization of the O-FMN patch and effect of the O-FMN patch on cellular behaviors. **a** Schematic of plasma surface modification using O_2_ plasma reaction gas on the surface of the FN patch. **b** SEM images of the O-FMN patch under various treatment times. The surface topography of the O-FMN patch showed the generation of nanopore structures without damage to the aligned nanotopography. Scale bars = 2 µm. **c** FT-IR analysis of the 30-min O-FMN. **d** XPS survey scans and **e** high-resolution O1s XPS spectra of FN patches and 30-min O-FMN patches. **f** Water contact angle measurements (*n* = 10 for each group) and wettability recovery (*n* = 5) on surfaces of the O-FMN patch. **g** Attachment and proliferation of cells on O-FMN patches (*n* = 6 for each group). After 5 days of cell culture, cell proliferation was higher on the 30-min O-FMN than on the 30-min N-FN. **h** Effect of O-FMN patches on the osteogenic mineralization of tenocytes. Alizarin Red staining revealed higher calcium expression levels on the 30-min O-FMN patches than on other samples and the tissue culture polystyrene substrate (TCPS). Quantification of osteogenic mineralization demonstrated the highest degree of osteogenesis by the cells on the 30-min O-FMN patches (*n* = 6 for each group). Scale bars = 200 µm. Error bars = mean ± standard deviation (*P < 0.05).

Figure 3a shows a schematic of the plasma treatment process used in this study (described in detail in “Methods”). SEM images of the surface morphology of the O-FMN patches revealed a highly aligned topography with grooves and ridges, similar to the well-organized topography of the tendon ECM (Fig. 3b). Nanosized pores generated by O_2_ plasma treatment for 30 min were observed due to etching and volatilization, which were not generated in the N-FN patches. The O-FMN patches showed the uniform distribution of various pore sizes and more pores, depending on the O_2_ plasma treatment time compared to the O-FF patches (Fig. 3b and Supplementary Fig. 1). The average pore sizes of 30 min O-FMN and 30 min O-FF patches were analyzed as 192.84 and 145.16 nm, respectively. These results are due to when oxygen plasma is treated on the flat surfaces of polymeric materials, the collision radius or sidewall collision of the reactive oxygen species occurs significantly less. In contrast, on the surface with nanotopography, the sidewall collision and the collision radius increase due to the diffusion of reactive oxygen species in the nanostructures, so that the bombardment and oxidizing effect occurs actively, forming numerous large pores^32^.

However, these pores did not result in notable deformation to the highly aligned nanotopography. This result indicated that the generation of nanosized pores onto the aligned nanotopography could form multiscale nanostructures similar to the complex microenvironment of the ECM. Thus, we hypothesized that the combination of multiscale nanostructures comprising nanopores and a well-defined nanotopography and functional groups applied by O_2_ plasma treatment would provide a physiochemically synergetic effect to improve cell affinity along with cell function and tissue regeneration.

To confirm whether the O_2_ reaction gas used in the plasma treatment process changed PCL properties, the chemical characteristics of the polymer were analyzed. The functional groups of the O-FMN patch were investigated by FT-IR spectroscopy (Fig. 3c). The characteristic absorption bands related to PCL (i.e., CH_2_ asymmetric stretching at 2944 cm^−1^, symmetric stretching at 2866 cm^−1^, C = O stretching vibration of carbonyl groups at 1721 cm^–1^, and deformation of C–O at 1161 cm^–1^) were detected in all samples, showing that functional groups were well maintained and were not affected by plasma treatments. The surface chemical composition of the O-FMN and FN patches was analyzed by XPS. As shown in Fig. 3d, all XPS spectra had three separated peaks corresponding to C1s (285 eV), N1s (400 eV), and O1s (532 eV). A distinct O1s peak at 532 eV in the O-FMN patch spectrum indicated that the O_2_ plasma had been successfully applied onto the FN patch. The surface atomic compositions of the FN patch were calculated to be 73.91%, 0.29%, and 25.81%, and those of the O-FMN patches were calculated to be 63.7%, 0.23%, and 36.07% for C1s, N1s, and O1s, respectively. The high-resolution XPS O1s spectra showed that the O1s peak of the FN and O-FMN patches can be decomposed into three components: C–O component (531.69 eV) and C = O (532.39 eV), and O = C–O (533.3 eV) components (Fig. 3e). The atomic configurations of the FN patch were calculated to be 11.7%, 6.83%, and 7.49% for C–O, C = O, and O = C–O, respectively. The atomic configurations of the O-FMN patch were calculated to be 10.29%, 13.48%, and 11.97% for C–O, C = O, and O = C–O, respectively. The wettability of the O-FF and O-FMN patches was evaluated by water contact angle measurement, showing that FN patches had a lower contact angle (82.56 ± 1.8°) than FF patches (88.32 ± 1.7°) (Fig. 3f). Similar to the N-FF and N-FN patch, the water contact angle of O-FF and O-FMN patches after plasma treatment were decreased, and 30-min O-FMN patches had a lower contact angle (18.4 ± 1.1°) than did 30-min O-FF patches (22.01 ± 2.2°). To confirm the hydrophobicity variation on the surface of O-FMN patches, the static contact angle measurements were conducted over O_2_ plasma treatment time (30 min, 1 h, 2 h, 4 h, 1 day, 2 days, and 6 days). As shown in Fig. 3f, O-FMN patches exhibited small hydrophobic recovery at 30 min to 4 h and substantial hydrophobic recovery at 1 day to 6 days after plasma treatment. However, the hydrophilicity of the O-FMN patch was well maintained compared with the high static water contact angle of FN patches. To confirm whether the generation of the pores on the scaffold surfaces affects the change in mechanical strength, the tensile strengths of the FF, FN, 30 min O-FF, and 30 min O-FMN patches were measured using a tensile tester and assessed (Supplementary Fig. 2). When a load was applied along the direction of the aligned topography, the FN patches with the aligned nanotopography exhibited slightly larger tensile stress (~10.38 MPa) than that (~9.62 MPa) of FF patches with the flat topography. However, the 30 min O-FF patches and the 30 min O-FMN patches with nanopores generated oxygen plasma treatment showed no significant difference compared to the FF patch and the FN patch, respectively (Supplementary Fig. 2). This trend was measured in breakpoint strain analysis of FF, FN, 30 min O-FF, and 30 min O-FMN patches.

### In vitro cell behaviors on O-FMN patches

Cell attachment and proliferation on N-FN and O-FMN patches were both higher than those on FF patches. In addition, after 5 days of cell culture, cell proliferation was considerably higher on O-FMN patches treated with O_2_ plasma for 30 min than that observed on 30-min N-FN patches (Fig. 3g). This suggests that the surface modified by O_2_ plasma may provide various cell-friendly functional groups and hierarchically topographical environments that are similar to the complex microenvironment of the ECM, thus promoting the proliferation and attachment of tenocytes when compared with the surface modified by N_2_ plasma. In addition, we examined the osteogenic mineralization of tenocytes on the N-FN and O-FMN patches by culturing cells in osteogenic induction medium for 14 days. Alizarin Red staining (Fig. 3h) revealed higher calcium expression levels on the O-FMN patches for 30 min than on N-FF and N-FN patches for 30 min and the TCPS. Quantification of osteogenic mineralization further demonstrated the highest degree of osteogenesis by cells cultured on the 30-min O-FMN patches. These results suggest that plasma treatment of FN patches may provide cell-friendly functional groups and topographical environments to enhance the attachment, proliferation, and differentiation of tenocytes. Therefore, the highly aligned nanotopography, generation of nanosized pores, and various functional groups induced by plasma treatment using O_2_ gas synergistically contributed to the proliferation and differentiation of tenocytes.

### In vivo animal study for soft- and hard-tissue regeneration

RC tendon tears are one of the most common causes of shoulder pain^33^. Surgical repairs of RC tendon tears have high re-tear rates, and thus many devices have been developed to augment the repair efficacy^34^. However, repairing defected mineralized fibrocartilage of the tendon–bone interface that causes the high re-tear rate of RC remains a challenge. Here, we propose an approach for the regeneration of mineralized fibrocartilage tissue of the tendon–bone interface. According to the results of in vitro analyses with tenocytes, we determined the optimum plasma treatment time and plasma reaction gas (O_2_ vs. N_2_) based on their effects on cell proliferation and differentiation. Since the O-FMN patch mimicking the multiscale ECM nanostructure with various functional groups could provide an efficient environment for cell proliferation and osteogenic mineralization, we hypothesized that the O-FMN patch with a highly aligned nanotopography, nanosized pore, and various functional groups would effectively guide RC tendon and mineralized fibrocartilage tissue regeneration. To confirm the tissue regeneration efficacy of the O-FMN patch, an acute RC tendon tear rat model was established.

**Fig. 4:**
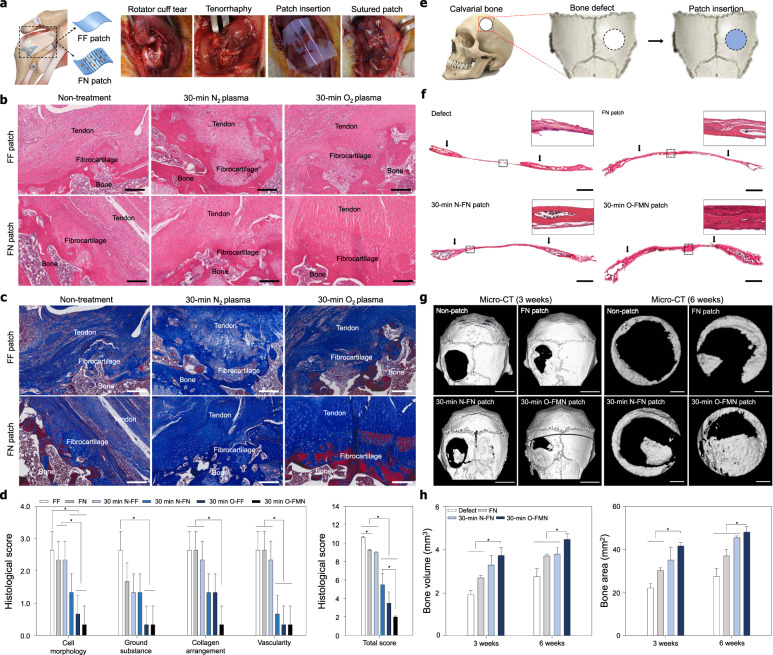
Effect of the O-FMN patches on the tendon and bone tissue regeneration. **a** Surgical procedure for RC tendon repair. All patches were grafted onto the defected tendon tissue after tenorrhaphy of the torn RC tendon (*n* = 3 for each group). **b** Representative histologic images of H&E staining and **c** Masson trichrome staining of the insertion site of FF, FN, 30-min N-FF, 30-min N-FN, 30-min O-FF, and 30-min O-FMN patches onto the supraspinatus tendon 4 weeks after repair. Scale bars = 200 µm. **d** Semiquantitative analysis of the histological evaluation scores on repaired tendon tissues of RC tear animal models. **e** Surgical procedure for rat calvarial bone repair (*n* = 5 for each group). **f** Representative histologic images of H&E staining of the insertion site of FN, N-FN, and O-FMN patches onto rat calvarial bone 6 weeks after repair. Scale bars = 1 mm. **g** Representative micro-CT image after 3 and 6 weeks of repair. Scale bars = 5 mm (3 weeks). Scale bars = 1 mm (6 weeks). **h** Quantitative analysis of bone volume and area of the bone regeneration site after 3 and 6 weeks of repair. Error bars = mean ± standard deviation (*P < 0.05).

Figure 4a shows the procedure of implantation of patches at the RC repair site (i.e., all samples were implanted after tenorrhaphy immediately following artificial tendon rupture). All rats survived to the designated sacrifice date, and no adverse events were observed. Hematoxylin and eosin (H&E) staining was performed to evaluate the histologic quality on the connection of collagen fibers in the tendon–bone interface, orientation and density of the collagen fibers, maturity of the tendon–bone interface, and cell confluency of the regenerated RC tendon and mineralized fibrocartilage tissue 4 weeks after repair with non-treated patches, 30-min N_2_ plasma-treated patches, and 30-min O_2_ plasma-treated patches (Fig. 4b and Supplementary Fig. 3). No infection, contracture, mobility disability, or inflammatory reaction were observed in any of the rats throughout the postoperative period. The tissues from rats treated with the N_2_ (N-FF and N-FN patches) and O_2_ (O-FF and O-FMN) plasma-treated patches showed native rat tendon tissue-like histological healing patterns (Supplementary Fig. 4), with high ratios of tendon tissue regeneration observed in the sections under the patches. The RC tendons repaired using the FN patches showed well-organized collagen fibers of high density, whereas those of the FF control groups showed sparse fibrous tissue and low cell confluence of the bone and fibrocartilage at the wound site (Fig. 4c). In addition, the tendon tissues treated with the N_2_ (N-FF and N-FN patches) and O_2_ (O-FF and O-FMN) plasma-treated patches showed organization of the collagen fibers similar to that of native tendon tissues, with increased amounts of cellular at the tendon–bone interface compared to those of non-treated groups. The fibrocartilage tissues at the tendon–bone interfaces treated with the O-FMN patch showed a more well-organized vertical arrangement compared to those of the non-treatment groups and N-FN patch groups. Importantly, the fibrocartilage tissues at the tendon–bone interfaces from rats treated with the O-FMN patch showed a vertical arrangement, alignment, and increased amounts of cellular similar to the native tendon tissue (Fig. 4c). The O-FMN patch had an obvious positive influence on the collagen organization, connection, and tendon tissue regeneration, resulting in a structure similar to that of native RC tendon tissues and fibrocartilage tissues at the tendon–bone interface. These results indicate the importance of a precisely aligned nanotopography and nanosized pore, which generated the multiscale structure of the native ECM microenvironment to effectively guide tendon tissue and fibrocartilage regeneration.

Based on the qualitative histological analysis of the effect of the O-FMN patch presented as a repair strategy for RC tendon tissue rupture, a semiquantitative histology analysis was performed using the Bonar scoring system (Fig. 4d and Supplementary Table 1). Cell morphology, ground substance, collagen arrangement, and vascularity changes were observed in all groups and were graded depending on Bonar score (Supplementary Table 2). The cell morphology and collagen arrangement scores of the O-FMN patch group were significantly lower than those of other groups (Fig. 4d). The ground substance and vascularity scores of the O-FMN patch group were similar to the score of the O-FF patch group but were significantly lower than those of other patch groups. Overall, the total histological score of the O-FMN patch group was significantly lower than those of other patch groups (Fig. 4d). These results indicate the importance of the oxygen plasma treatment, a precisely aligned nanotopography, and the nanoporous structures in synthetic ECMs for guiding tendon tissue and fibrocartilage regeneration.

In addition, we confirmed the effects of N_2_ and O_2_ plasma treatment and nanotopograhpy throughout bone regeneration in vivo (Fig. 4e). All mice used in the in vivo studies survived to the date of sacrifice, no adverse events were observed. FN patch, N-FN and O-FMN patch were engrafted onto the calvarial bone defect with 5 mm diameter (Fig. 4e). No infection or inflammatory response was observed in any mice during the postoperative period. The implanted patches were maintained for 6 weeks without deformation. To confirm the bone regeneration efficacy from N_2_ and O_2_ plasma treatment and nanotopograhpy, we conducted the H&E staining at 6 weeks after implantation (Fig. 4f). As the result, the bone of the defect groups was empty in the defect area. More bone formation and dense cytoplasm occurred in O-FMN patches compared to other groups were confirmed.

To quantitatively evaluate the effects of nanotopography and plasma treatment on bone formation, we performed micro-CT and 3D-image conversion using the MIMICS 14.0 software on new bone defects in vivo. As shown in Fig. 4g, formation of bone by the nanotopographic and plasma-treated patches occurred along the periphery of the bone defect and grew along the patches. After 3, 6 weeks, the compact bone formation was not observed in the defect group, FN patch groups, and N-FN groups whereas bone regeneration was significantly enhanced in the O-FMN patch groups after 3, 6 weeks of implantation. The new bone formation was observed from edge to center depending on nanotopograhpy direction. At the 6 weeks, the bone volume was 1.91 mm^3^ in the defect group, 2.43 mm^3^ in the FN patch groups, and 3.38 mm^3^ in the N-FN patch groups, and 4.25 mm^3^ in the O-FMN patch groups (Fig. 4h). The bone area was 19.81 mm^2^ in the defect groups, 26.09 mm^2^ FN patch groups, 37.92 in the N-FN patch groups, and 47.92 mm^2^ in the O-FMN patch groups (Fig. 4h). The results of bone regeneration and formation provide insight into the importance of nanotopography and O_2_ plasma treatment cues for inducing hard-tissue regeneration.

### Quantitative investigation of relative contributions

Based on these in vitro and in vivo results, we quantified the capability of tissue regeneration and cell function enhanced by the beneficial effects of the O-FMN patch on proliferation and differentiation (Supplementary Fig. 5). Quantitative investigation of relative contributions was derived by setting the raw average values of the proliferation and osteogenic mineralization absorbance of the FF patch as 1, and calculating relatively the absorbance of the FN, 30-min N-FF, 30-min N-FN, 30-min O-FF, and 30-min O-FMN patches. The aligned nanostructure of the FN patch, functional groups applied by N_2_ plasma treatment, and nanosized pores and functional groups generated by the O_2_ plasma treatment could only promote proliferation by a factor of 1.01, 1.12, and 1.1, respectively, and osteogenic differentiation was promoted by a factor of 1.37, 1.32, and 1.44, respectively. The combination of a nanotopography and N_2_ plasma treatment increased proliferation and osteogenic differentiation by a factor of 1.36 and 1.4, respectively. In addition, the combination of nanotopography and O_2_ plasma treatment significantly increased the proliferation and osteogenic differentiation by a factor of 1.74 and 1.78, respectively, further confirming the enhanced effects of highly aligned nanotopography, nanosized pores, and functional groups. This synergetic effect may be due to improvement in cell topography or cell–cell interactions from the highly aligned nanotopography and nanosized pores generated by O_2_ plasma treatment.

## Discussion

Developing a functional scaffold is crucial for effectively replacing, repairing, or engineering human cells, tissues, and organs to restore or establish normal biological function^4,9,35^. In particular, a transplantable scaffold should be designed to allow for control of the mechanical properties, architectures, structures, biodegradability, and biocompatibility to suit the purpose for regenerating the function of the tissues or organs of interest. Based on the key consideration of designing a transplantable scaffold, we developed flexible multiscale nanotopographic patches (O-FMN) inspired by the in vivo-like microenvironment of the ECM of tendon tissue by employing CFL and plasma treatment using the FDA-approved PCL polymer, which possesses advantageous properties of biocompatibility and biodegradability. Thus, we considered four major factors for designing an efficient scaffold as a transplantable platform: (i) flexibility with respect to mechanical properties, (ii) topographical properties, (iii) biodegradability, and (iv) biocompatibility.

(i) Flexibility with respect to mechanical properties: The mechanical properties of a scaffold should be designed to match with the implantation site tissues or should be sufficient to shield cells from destructive compressive or tensile forces without inhibiting suitable biomechanical signals while allowing for persistence under physiological conditions^36,37^. In particular, achieving flexible bio-integrated scaffolds against the highly soft and irregularly shaped surfaces of human tissues has been a primary goal for diagnostic and therapeutic capabilities^16,38^. Accordingly, using a PCL polymer, we developed a patch-type flexible substrate for transplantation in human tissue prior to the implementation of multiscale nanotopography onto the PCL-based flexible substrate. PCL polymer is one of the major synthetic polymers used for the fabrication of scaffolds as biomaterials in the biomedical field owing to its good mechanical strength, extremely high elongation potential, and suitable flexibility depending on the thickness of the scaffold^39–41^. Therefore, we assert that PCL-based flexible multiscale nanotopographic (O-FMN) patches provide the ability to intimately contact the curvilinear, actively moving surface of living biological tissues such as the skin, muscles, cartilage, and tendon because of the thin and flexible properties.

(ii) Topographical properties: In the ECM microenvironment, various cells are exposed to very complex and controlled topographical signaling cues with different combinations of micro- and nanoscales such as aligned collagen structures, mesh collagen structures, and the porous structure of the ECM in various tissues^11,42^. In conjunction with these aspects, previous studies have described various artificial ECM platforms inspired by the native ECM microenvironment, which have unique structural and topographical properties, such as the bone^17,25,43,44^, skin^7,45^, muscle^35,46^, and tendon^27,47–49^. Consequently, these scaffolds designed in compliance with the multiscale topographical properties of the ECM appear to be crucial to control and improve the morphology, migration, proliferation, differentiation, and metabolism of cells. In conjunction with these aspects, we developed a methodology to fabricate a multiscale scaffold with a highly aligned nanotopography in combination with a nanoporous structure inspired by the complex topographical features of the natural ECM. First, to fabricate the highly aligned nanotopography, we used CFL technology, which can easily control the micro- or nanotopography sizes and structures with scalable and highly reproducible properties using various biomaterials as well as polymeric materials^17,50,51^. Second, to fabricate the nanoscale porous structure onto this highly aligned nanotopography, we employed O_2_ plasma surface treatment, which can structurally modify the polymeric surface owing to the volatility of chemical bonding^52–54^. Various reaction gases such as nitrogen, oxygen, argon, and air have been used in plasma treatment for polymeric surface modification^55^. Among the various gases for the surface plasma modifications of biomaterials, O_2_ plasma surface modification has been proven as an effective and inexpensive strategy to alter physicochemical, mechanical, and biological properties such as roughness, wettability, hardness, and biocompatibility^56^. The oxygen plasma induces pore formation by etching the polymer surface through the reaction of surface carbon atoms with various oxygen species in the plasma, including electrons, ions, radicals, and neutral molecules, to produce volatile reaction products such as H_2_O and CO_2_. The formation of pores is due to the impact and oxidation effects on the polymer surface induced by energetic ions and radicals present in the oxygen plasma^57^. Although both of the oxygen and nitrogen gas plasma introduce chemical groups to the polymer surface, the nitrogen gas plasma is not responsive enough to generate porous structures of the surface of the PCL scaffolds because it has significantly lower reactivity with carbon atoms than oxygen gas plasma^58^. In addition, O_2_ plasma has been used to produce carboxyl, carbonyl, and hydroxyl functional groups altering surface chemistry and increasing hydrophilicity onto the surface of various polymers to improve cell attachment and strength of adherence to these plasma-treated surfaces cell attachment, proliferation, and differentiation^59^. Thus, we suggest an efficient design methodology combining CFL and oxygen plasma treatment to obtain a scaffold with multiscale nanotopography for creating native ECM-like structures for advanced applications of tissue engineering and regenerative medicine.

(iii and iv) Biodegradability and biocompatibility: In this study, we selected the PCL polymer to design the transplantable scaffolds due to its suitability for tissue regeneration and wide applications in the biomedical engineering. PCL is one of the most widely used polymers for fabricating biomedical scaffolds due to its controllable biodegradability within months to years depending on the molecular weight, crystallinity of the polymer, and degradation conditions^17,60–66^. PCL has the ability to promote the formation and growth of new tissues, and its performance degrades when sufficient tissue regeneration occurs, while supporting and disposing of defects, making it a suitable material for replacing or repairing damaged tissue. Furthermore, since PCL is relatively inexpensive in addition to being already approved by the US FDA, scaffolds based on PCL polymer could be more easily commercialized than those based on other biomaterials. Thus, PCL is one of the ideal materials for developing the implantable scaffolds for tissue regeneration or replacement of a defective or damaged organ. However, although PCL has biocompatibility and biodegradability, its low bioactivity (associated with the absence of bioactive chemical groups on its surface) and low surface energy clearly reduce cell affinity and inhibits cell interactions, thereby slowing tissue regeneration^64^. Therefore, the use of synthetic polymers (including PCL) for the development of efficient scaffolds for tissue engineering must overcome these limitations. To address this issue, we used plasma treatment to enhance cell affinity and bioactivity to fabricate surface-modified hydrophilic scaffolds. Plasma modification can improve the hydrophilicity of polymers by inducing specific functional groups on the surface, thereby altering the chemical signature, wettability, and energy without altering the bulk properties. In addition, the plasma treatment has efficient properties to change the chemistry and structures of the surface with high control without additional solvents, allowing for reducing the chemical reagents used and their intrinsic sterility due to the presence of highly energetic species^67^.

Comprehensively, as a design methodology to fabricate a transplantable scaffold, we propose several strategies to improve bioactivity and biofunctionality. PCL-based FMN patches have the ability to intimately contact a curvilinear surface. Inspired by the complex multiscale structure and nanotopographical features of the native ECM, a material with a multiscale topography, and a nanoporous and aligned nanotopography were constructed onto flexible PCL substrates using CFL technology and O_2_ plasma etching for enhanced cellular functions and behaviors in terms of architectural properties. To overcome the limitations with respect to cell affinity and bioactivity due to the hydrophobic property of PCL, the PCL substrates were treated with N_2_ or O_2_ plasma, thereby inducing certain functional groups on the surface.

Our in vitro study yielded three interesting findings in terms of the highly aligned nanotopographical cue and nanoporous structure of the multiscale structures along with the effects of the plasma treatment on cellular behaviors. First, the highly aligned nanotopographical cues improved osteogenic mineralization. Second, N_2_ plasma treatment for 30 min onto the highly aligned nanotopographical patch and flat patch enhanced cell proliferation. In particular, the combination of N_2_ plasma treatment for 30 min and the highly aligned nanotopographical cues significantly improved osteogenic mineralization. Based on these findings, we hypothesized that plasma treatment for 30 min can maximize the efficiency of cellular behaviors. Third, O_2_ plasma treatment for 30 min generated nanopores, and the multiscale nanotopographical cues with the aligned nanotopography and nanoporous structures considerably improved cell proliferation as well as the osteogenic mineralization of cells. Consequently, in vitro study showed that the O-FMN patch with an aligned nanotopography and nanoporous structure, along with additional chemical functionality by plasma treatment provides a suitable environment for cell growth and function. Based on our observation on proliferation and osteogenic mineralization, we propose the following possible mechanism for the enhancement on a flexible multiscale nanotopographic patch (Supplementary Fig. 5):

1. Enhanced cellular behaviors from the nanotopography, based on (i) the enhanced proliferation factor [from 1 (FF patch) to 1.01 (FN patch)] and (ii) enhanced osteogenic mineralization factor [from 1 (FF patch) to 1.37 (FN patch)].
2. Enhanced cellular behaviors from plasma treatment, based on (i) the enhanced proliferation factor [from 1 (FF patch) to 1.12 (N-FF patch) and from 1 (FF patch) to 1.1 (O-FF patch)], and (ii) enhanced osteogenic mineralization factor [from 1 (FF patch) to 1.32 (N-FF patch) and from 1 (FF patch) to 1.44 (O-FF patch)].
3. Enhanced cellular behaviors due to the combination of nanotopography and plasma treatment, based on the (i) enhanced proliferation factor [from 1.01 (FN) to 1.36 (N-FN patch) and from 1.01 (FN patch) to 1.74 (O-FMN patch)], and (ii) enhanced osteogenic mineralization factor [from 1.37 (FN patch) to 1.4 (N-FN patch) and from 1.37 (FN patch) to 1.78 (O-FMN patch)].

Using the O-FMN patch, we demonstrated that the complex ECM-like multiscale nanotopography feature could promote the tissue regeneration of the mineralized fibrocartilage of the tendon–bone interface and calvarial bone. This finding was due to the enhanced cell proliferation and osteogenic mineralization owing to multiscale nanotopography cues with an aligned nanotopography and nanoporous structure. The aligned nanotopography and porous properties of the patch can be more efficiently applied to the complex microenvironment of the mineralized fibrocartilage and bone compared to a single scale nanotopography design. Furthermore, these structural properties in the living cell environments play an important role in providing an active nutrient supply as well as enabling enhanced tissue bonding and precise cell-topography interactions that are required to guide cells toward the nanotopographical cue for reconstructing tissue in a manner similar to native tissue formation. Moreover, the flexible and hydrophilic properties of flexible multiscale nanotopographic patches provided compact adaptation to the curved tissue surface of the tendon–bone interface and bone for easy handling during surgical procedures. Thus, the mechanical and structural properties of the O-FMN patch might improve cellular functions and behaviors to eventually promote tendon and bone tissue regeneration.

The in vitro and in vivo experiments demonstrated the effect of multiscale nanotopographic cues with an aligned nanotopography and nanoporous structure on the enhanced behaviors and functions of cells to promote tissue regeneration. On the basis of our study, we propose other potential biomedical applications of this PCL-based O-FMN patch. For example, this scaffold or technology may allow for the fabrication of precisely defined complex multiscale (micro- or nanosized) substrates for tissue regeneration in various organs (e.g., the skin, nerve, tooth, muscle, and heart). Recently, stem cell-based therapy has been highlighted as another promising strategy for maximizing the regeneration of various tissues^68–70^. Accordingly, our O-FMN patch could be integrated with a stem cell platform to obtain better control and enhancement of stem cell behaviors and functions as well as to prevent their loss in the targeted tissue area. Moreover, this platform of a flexible patch type can be used to design and fabricate transplantable biomaterials that require flexibility, such as artificial blood vessels, dental membranes, wound dressings, and artificial electronic skin, given the ability for compact adaptation to the underlying tissue. Overall, this study provides new insight into the design of efficient multiscale topography-based scaffolds for various biomedical applications.

In this study, we tried to show the potential for the effectiveness of the novel scaffolds for soft- and hard-tissue regeneration in rotator cuff tear and cranial defects in animal models. (Fig. 4). Since we demonstrated the important evidence on the functions of scaffold (e.g., morphological analysis and histological evaluation) in this study, especially for clinical applications, detailed in vivo studies (e.g., large animal models, biomechanical tests, and physiological functions, etc.) should be performed using the proposed scaffolds.

In conclusion, A hierarchical multiscale patch can be used as an efficient methodology for the design and manipulation of scaffolds with a flexible multiscale topography to enhance cellular behaviors and tissue regeneration. FMN patches with precisely controlled hydrophilic properties and hierarchical nanostructures of the surface were fabricated via CFL and plasma surface modification using a PCL polymer. We propose that a hierarchical multiscale scaffold comprising nanotopographic and chemically modified surface cues could provide a native ECM-like physicochemical microenvironment for controlling the proliferation and osteogenic mineralization of cells. N_2_ or O_2_ plasma-assisted multiscale nanotopographic scaffolds showed good potential for regeneration of the tendon, the mineralized fibrocartilage, and bone tissue. Consequently, our quantitative investigation collectively demonstrated that combinations of an aligned nanotopographic, nanoporous structure, and chemically modified surface cues could enhance effects on cellular behaviors. Our study provides insight into the design and manipulation of a flexible multiscale topography with controlled hydrophilic properties for promoting soft- and hard-tissue regeneration.

## Methods

### Design and fabrication of a flexible nanotopographic (FN) patch

Because of the surface charge of the silicon wafer, the nanotopographical PCL patch imprinted directly on the silicon wafer was not easily peeled off. Therefore, we used the PDMS mold, whose surface is a negative charge, to easily peel off the PCL patches. In addition, heat and pressure applied in the fabrication process to fabricate the nanotopographical PCL patch can easily cause damage to the silicon wafer master mold. This limitation does not guarantee reproducibility to fabricate a large number of the nanotopographical PCL patches for various experiments. Therefore, to maintain reproducibility of the original nanostructure by minimizing damages, a mother molds were fabricated with PDMS and PUA, and then the nanotopographical PCL patches were indirectly fabricated using it.

The detailed methods have already been reported by our group^27^. A droplet of ultraviolet (UV)-curable polyurethane acrylate (PUA) (Changsung Sheet., Korea) precursor solution with a photoinitiator was dropped onto a silicon master mold, on which nanosized (800 nm) linear grooves and ridges were etched using conventional photolithography and reactive ion etching. The mold was then uniformly covered with a transparent poly (ethyleneterephthalate) (PET; SKC, Korea) film utilizing capillary force. After the master mold was exposed to UV light (*λ* = 352 nm, 40 w) for 60 s, the cured nanopatterned PUA replica was peeled off from the master mold using tweezers and again exposed to UV light overnight to eliminate any residual reactive acrylate groups. First, the nanopatterned PUA mother mold was attached to a Petri dish with the nanotopographic surface facing up. A polydimethyl siloxane (PDMS) pre-polymer (Sylgard 184 Silicon elastomer, Dow Corning, USA) was mixed with a 10% curing agent, poured onto the nanopatterned PUA mother mold in a Petri dish to a sufficient thickness (~1 cm), and baked at 70 °C for at least 6 h to ensure curing without any residue formation. The cured nanopatterned PDMS mold (800-nm ridges and grooves) was then peeled off from the PUA mother mold in the Petri dish. To fabricate the flexible flat (FF) patch as a control for comparison, a flat silicon wafer was attached to the Petri dish with the flat surface facing up. Similar to the fabrication protocol of the nanopatterned PDMS mold, the same PDMS pre-polymer was mixed with the 10% curing agent, poured onto the flat silicon wafer in the Petri dish to a sufficient thickness (~1 cm), and baked at 70 °C for at least 6 h to ensure curing without any residue formation. The cured flat-patterned PDMS mold was then peeled off from the flat silicon wafer in the Petri dish.

PCL pellets (Mw: 80,000; Sigma-Aldrich, USA) were dissolved in dichloromethane using a magnetic stirrer, and a PCL solution of 18 wt% (wt/wt) in dichloromethane (Daejung Chemicals & Materials Co., Ltd, Korea) was prepared. A thin PCL patch was fabricated by spin-coating the PCL solution that was poured into a 1.5-mm circular glass on the vacuum cuck of the spin coater. The spin-coating condition was as follows: rotator speed of 3500 rpm, duration of 120 s, and acceleration time of 5 s. First, we fabricated the FF patch as a control group because the surface of the spin-coated PCL patch has irregular roughness that limits the fabrication of an FN patch. The fabricated thin PCL patch was placed onto the silicon wafer substrate face-up to melt the PCL layer on a hot plate for 60 s at 80 °C. The flat-patterned PDMS mold was placed and embossed onto the pre-melted PCL layer by applying pressure with smooth finger force while heating at 80 °C for 2 min. After the thermal imprinting process, the assembly of the PCL layer on circular glass and PDMS molds was cooled at 25 °C for 30 min, and the PDMS mold was peeled off from the PCL layer on the circular glass, resulting in an FF patch. And then, FF patches were used to fabricate FN patches. The fabrication process of the FN patch is almost similar to that of FF patch, except that it uses a nanopattern PDMS mold. In this study, all patches were separated from the circular glass by washing with 70% ethanol for implantation in the in vivo study.

### Plasma modification

The fabricated FF and FN patches were washed with ethanol and dried. Surface treatment of patches was carried out using a CUTE-1MP (Femto Science, Korea) low-pressure plasma system. Before processing, the empty chamber was cleaned for 5 min with O_2_ gas plasma (30 W generation power, 60 sccm gas flow rate, and 5.52e^−1^ Torr pressure). N_2_ and O_2_ gas plasma treatments were performed for 1 min, 5 min, 10 min, 20 min, and 30 min each under 30 W generation power, 60 sccm gas flow rate, and 5.52e^−1^ Torr pressure.

### Characteristics and properties analysis

N-FF and N-FN patches treated for 0, 1, 5, 10, 20, and 30 min were analyzed, and O-FF and O-FN patches treated for 0 and 30 min were analyzed using a high-resolution field-emission scanning electron microscope (FE-SEM), Fourier transform-infrared spectroscopy (FT-IR), and X-ray photoelectron spectroscopy (XPS). FE-SEM images of the surface of all patches fabricated in this study were observed using a JSM-7500F microscope (Oxford, UK) at an acceleration voltage of 15.0 kV and an average working distance of 8.8 mm. The samples were coated with platinum prior to morphological observation. Chemical characteristics of plasma-treated flat patches and plasma-treated nanotopographic patches were analyzed to confirm their chemical variation. The chemical bond structures were examined by FT-IR (Spectrum 400, USA). The surface chemical composition was analyzed using XPS (K-ALPHA + , Thermo Scientific, USA). The XPS survey spectra were recorded using a monochromatic Al Kα source (1486.67 eV) with a spot size of 200 µm and an electron take-off angle of 90°. The typical base pressure was below 2 × 10^−9^ mbar. Survey spectra were recorded in the range of 0–1350.0 eV with a pass energy of 200 eV, step size of 1.0 eV, and dwell time of 10.0 ms. The mechanical properties (i.e., stress and strain) of the patches (width: 12 mm and length: 20 mm) were measured using an MCT-1150 tensile tester (A&D Company, Japan) at a crosshead speed of 100 mm/min. The samples were analyzed by applying load along the direction of aligned nanotopography. The sample was measured at a crosshead distance of 10 mm from the center of the sample.

### Water contact angle measurements

The static water contact angle of liquids was measured using customized camera systems with a Computer M1214-MP2 2/3” Fixed Lens and analyzed using the ImageJ software. N-FF and N-FN patches treated for 0, 1, 5, 10, 20, and 30 min were analyzed, and O-FF and O-FN patches treated for 30 min were analyzed. For each measurement, 10 μL of water was dropped onto the surface or a period of 1 min. The water contact angle was measured as a tangent to the droplet interface of the patch. Measurements were repeated and averaged at least five times for each sample. All experiments were performed at room temperature.

### Isolation and culture of tenocytes

The detailed methods have already been reported by our group^27^. The tendon tissue samples were collected from the supraspinatus tendon of patients during arthroscopic rotator cuff (RC) repair surgery after obtaining informed consent from patients at Chonnam National University Medical School and Chonnam National University Hospital (CNUH-2013-065). The tendon tissue samples were washed with phosphonate-buffered saline (PBS; Sigma-Aldrich, USA), cut into small pieces, and digested with 3 mg/ml collagenase (Sigma-Aldrich, USA) in Dulbecco’s modified Eagle’s medium (DMEM; Cellgro, USA) at 37 °C for 16 h. After enzymatic digestion, equal volumes of DMEM were added to quench the collagenase and filtered through cell strainers (70-μm mesh). The filtered cell suspension was centrifuged at 1500 rpm for 5 min. The cell pellets were resuspended and cultured in DMEM low-glucose (Cellgro, USA) supplemented with 10% fetal bovine serum (FBS; Cellgro, USA)) and 1% penicillin-streptomycin (GenDEPOT, Houston, TX, USA) at 37 °C in a 5% CO_2_ atmosphere. The medium was changed every 3 days. All cells used in this work were at passages 4–6.

### Cell attachment and proliferation analysis

Tenocytes (1 × 10^4^ cells/samples) were seeded onto the samples and cultured for 6 h, 3 days (cell proliferation), and 5 days in DMEM containing 10% FBS and 1% antibiotics (Cellgro, USA) at 37 °C in a humidified atmosphere containing 5% CO_2_. Quantitative analysis of cell proliferation on the samples was performed using a WST-1 assay (Premix WST-1 Cell Proliferation Assay System, Takara Bio Inc., Kusatsu, Japan). To confirm cell attachment, the patches were washed using PBS to remove any cells not attached to the scaffolds prior to quantitative analysis using the WST-1 assay.

### Osteogenic mineralization analysis

Tenocytes (4 × 10^4^ cells/sample) were cultured for 14 days on samples in osteogenic differentiation medium (100 nM dexamethasone, 50 μM ascorbic acid, and 10 mM glycerol 2-phosphate in normal media). Alizarin Red S (Sigma-Aldrich, USA) staining was used to confirm the osteogenic differentiation (according to the degree of mineralization) of tenocytes on sample surfaces. The stained cells were de-stained with cetylpyridinium chloride (Sigma-Aldrich, USA), and the extracted solutions were measured using an absorbance reader (iMarkTM Microplate Absorbance Reader, Bio-Rad, Hercules, CA, USA) at 595 nm to quantify the osteogenic differentiation of tenocytes.

### In vivo animal study

The detailed methods have already been reported by our group^27^. The animal study was approved by the Ethics Committee of Chonnam National University Medical School and Chonnam National University Hospital (CNUHIACUC-18013). Sprague-Dawley rats weighing 350–450 g per group were equally divided into the FF patch group, FN patch group, N-FF patch group, N-FN patch group, O-FF patch group, and O-FMN. The N-FF, O-FF, N-FN, and O-FMN patches treated for 30 min each were used for animal study. The rats were fully anesthetized with an intramuscular injection of 35 mg/kg ketamine (Youhan Corporation, Seoul, Korea) and 5 mg/kg xylazine hydrochloride (Rompun; Bayer HealthCare, Korea). Both shoulders of each rat were shaved and disinfected with povidone-iodine (Firson, Korea), and the animals were placed in a lateral position with the forelimbs in adduction and external rotation. A 2.0-cm skin incision was made over the scapulohumeral joint, subcutaneous tissues were dissected, and the omotransverse and trapezius muscles were retracted to expose the supraspinatus tendon (located superior to the scapular spine). The acute RC model was established by the sharp release of the supraspinatus tendon at the greater tuberosity of the humerus over a 5 mm width, and surgical RC repair was performed immediately after creating the supraspinatus tendon tear. The torn supraspinatus tendon was repaired with 2.0 Ticron (Tyco, Waltham) in a transosseous manner after creating a bleeding bed at the footprint of the greater tuberosity. Two bone tunnels were created at the articular margin of the footprint to the lateral humeral cortex. The suture was passed through the bone tunnels and tied, and the supraspinatus tendon was reattached to the footprint. The FF patch (*n* = 3), FN patch (*n* = 3), N-FF patch (*n* = 3), N-FN patch (*n* = 3), O-FF patch (*n* = 3), and O-FMN patch (*n* = 3) were used to augment the repair site by stitching the proximal portion of the patch to the supraspinatus tendon and the distal portion of the patch to the soft tissue at the lateral portion of the proximal humerus. All patches (dimension: 16 × 24 mm) were well adhered to the defect site of supraspinatus tissue and sufficiently covered on the tendon to the bone interface of the rotator cuff. The fascia and subcutaneous tissues were sutured using interrupted 3–0 vicryl sutures (Ethicon, Johnson and Johnson, USA), and the skin was sutured with interrupted 4–0 prolene sutures. All rats tolerated this procedure without any intraoperative complications. After surgery, analgesis (Trodon Inj, 1 mg/ml, Aju Pharm, Korea) and antibiotic (Baytril 50 inj. (enrofloxacin), 1 mg/ml, Bayer, Korea) were administered by intramuscular injection. The shoulders were not immobilized postoperatively. Rats were housed individually and had free access to water and food. The rats were sacrificed 4 weeks after surgery to obtain tissues including the tendon, fibrocartilage, and bone regions of the rotator cuff.

In addition, the animal study was approved by the Ethics Committee of Chonnam National University. 6-week-old, male mice (C57Bl/6N) were assigned into four groups of four each: Defect, Nano, N-FN patch, and O-FMN. The mice were fully anesthetized with an intraperitoneal injection zoletil 0.006 cc/10 g and rumpun 0.004 cc/10 g, the heads were shaved and disinfected. The bones were exposed by incising the skin approximately 3.0 cm above the calvaria bone. The bone defects (diameter: 5 mm) were made on one side of the revealed calvarial bone using an electric drill. Prepared patches (Diameter: 5 mm) were placed on the calvarial bone defect. After suturing the skin with sutures, the ambient temperature was raised, and mice were waked up from anesthesia. The mice were sacrificed 3 and 6 weeks after surgery to obtain tissues including the defect region and the calvarial bone.

### Histological observations and evaluation

The detailed methods have already been reported by our group^27^. The proximal humerus including the greater tuberosity head with attached supraspinatus tendon of both shoulders of each rat was harvested. Specimens were fixed in neutral buffered 10% formalin (pH 7.4) and decalcified with Calci-Clear Rapid (National Diagnostics, Atlanta) for 2 weeks, and paraffin blocks were made in the repair site including supraspinatus tendon and greater tuberosity. Sections (4 μm thickness) were cut in the coronal plane and stained with H&E and Masson’s trichrome. We assessed cellularity, collagen fiber continuity, orientation, density, and maturation of the tendon to the bone interface, and we also evaluated the inflammation rate around the patch at the tendon-to-patch interface. Images were captured and acquired using an Aperio ImageScope (Leica, CA, USA) software. General histological evaluation was performed with hematoxylin and eosin (×200 magnification), Masson’s trichrome (×200 magnification), and Picrosirius red (×100 magnification) stained slides of chronic RC tear animal models. The slides were evaluated using the semiquantitative grading scale of Bonar score, which assesses four variables (cell morphology, ground substance, collagen arrangement, and vascularity) of the tendon to bone interfaces. A four-point scoring system is used, where 0 indicates a normal appearance and 3 a markedly abnormal appearance (Supplementary Table 1)^71–73^. The total histological scores for each group were calculated from the sum of these four characteristic grades. Four sections were randomly selected from each group and were evaluated blindly by three independent assessors. The average score was used for comparison. The calvarial bone tomography was performed using Skyscan001172 (Skyscan, Konitch, Belgium) micro-computed tomography (Micro-CT) at a resolution of 11.38 pixels and exposure time of 316 ms, with an energy source of 80 kV and current of 124 µA. An average of 488 slices of calvarial bone was scanned. The Micro-CT images were analyzed using MIMICS 14.0 3D imaging software (Materialise’s Interactive Medical Image Control System, Leuven, Belgium). The calvarial bone specimens were fixed in 10% formalin and decalcified in a 0.5 M EDTA (pH 7.4) solution at room temperature for 7 days. After the specimens were embedded in paraffin, cut into 5-µm-thick sections. And then, they were stained with H&E. Images were obtained by the Aperio Images Scope (Leica, CA, USA) software.

### Statistical analysis

All quantitative data are presented as the mean ± standard deviation. Unpaired Student’s *t* tests were used for the statistical analysis of the cell adhesion, viability, and differentiation results. To compare three or more conditions, a one-way ANOVA was performed. *P* value of less than 0.05 was statistically significant. Statistical analyses of the Micro-CT were performed using Kruskal−Wallis testing with SPSS software.

### Reporting summary

Further information on research design is available in the Nature Research Reporting Summary linked to this article.

## Supplementary information

Supplementary Information

Reporting Summary

## Data Availability

All data that support the findings of this study are available from the corresponding author upon reasonable request.
