# Supplementary material for: Plasma-assisted multiscale topographic scaffolds for soft and hard tissue regeneration
Source: NPJ Regen Med. 2021 Sep 9;6:52. doi: 10.1038/s41536-021-00162-y (PMC8429553; doi:10.1038/s41536-021-00162-y)
Supplement: Supplementary file 1 — Supplementary Information [file 41536_2021_162_MOESM1_ESM.pdf]

Supplementary Information

Plasma-assisted multiscale topographic scaffolds for soft and hard tissue regeneration

Woochan Kim[1, 2, +], Yonghyun Gwon[1, 2, +], Yang-Kyung Kim[3], Sunho Park[1, 2], Sung-Ju Kang[3], Hyeng-Kyu Park[4], Myung-Sun Kim[3, *], Jangho Kim[1, 2, *]

[1] Department of Rural and Biosystems Engineering, Chonnam National University, Gwangju 61186, Republic of Korea
[2] Interdisciplinary Program in IT-Bio Convergence System, Chonnam National University, Gwangju 61186, Republic of Korea
[3] Department of Orthopedics, Chonnam National University Hospital, Gwangju 61649, Republic of Korea
[4] Department of Physical and Rehabilitation Medicine, Chonnam National University Medical School & Hospital, Gwangju 61649, Republic of Korea

[*] Corresponding authors: Correspondence should be addressed to Myung-Sun Kim (mskim@chonnam.ac.kr) or Jangho Kim (rain2000@jnu.ac.kr)

[+] These authors contributed equally to this work.

Variables	Grade			
	0	1	2	3
Cell morphology	Inconspicuous elongated spindle-shaped nuclei with no obvious cytoplasm at light microscopy	Increased roundness: nucleus becomes more ovoid to round in shape without conspicuous cytoplasm	Increased roundness and size: the nucleus is round, slightly enlarged and a small amount of cytoplasm is visible	Nucleus is round, large with abundant cytoplasm and lacuna formation (chondroid change)
Ground substance	No stainable ground substance	Stainable mucin between fibers but bundles still discrete	Stainable mucin between fibers with loss of clear demarcation of bundles	Abundant mucin throughout with inconspicuous collagen staining
Collagen arrangement	Collagen arranged in tightly cohesive well-demarcated bundles with a smooth dense bright homogeneous polarization pattern with normal crimping	Diminished fiber polarization: separation of individual fibers with maintenance of demarcated bundles	Bundle changes: separation of fibers with loss of demarcation of bundles giving rise to expansion of the tissue overall and clear loss of normal polarization pattern	Marked separation of fibers with complete loss of architecture
Vascularity	Inconspicuous blood vessels coursing between bundles	Occasional cluster of capillaries, less than one per 10 high-power fields	1–2 clusters of capillaries per 10 high-power fields	Greater than two clusters per 10 high-power fields

Supplementary Table 1. Histological evaluation grade (Bonar score) to assess cell morphology, ground substance, collagen arrangement, and vascularity of repaired tendon to bone interface.

Variables	FF patch				FN patch				N-FF patch				N-FN patch				O-FF patch				O-FMN patch			
	0	1	2	3	0	1	2	3	0	1	2	3	0	1	2	3	0	1	2	3	0	1	2	3
Cell morphology	0	0	1	2	0	0	2	1	0	0	2	1	0	2	1	0	1	2	0	0	2	1	0	0
Ground substance	0	0	1	2	0	1	2	0	0	2	1	0	0	2	1	0	2	1	0	0	2	1	0	0
Collagen arrangement	0	0	1	2	0	0	1	2	0	0	2	1	0	2	1	0	0	2	1	0	2	1	0	0
Vascularity	0	0	1	2	0	0	1	2	0	0	2	1	1	2	0	0	2	1	0	0	2	1	0	0

Supplementary Table 2. Distribution of histologic scores on repaired tendon to bone interface of RC tendon tear animal models using histological evaluation grades (Bonar score).

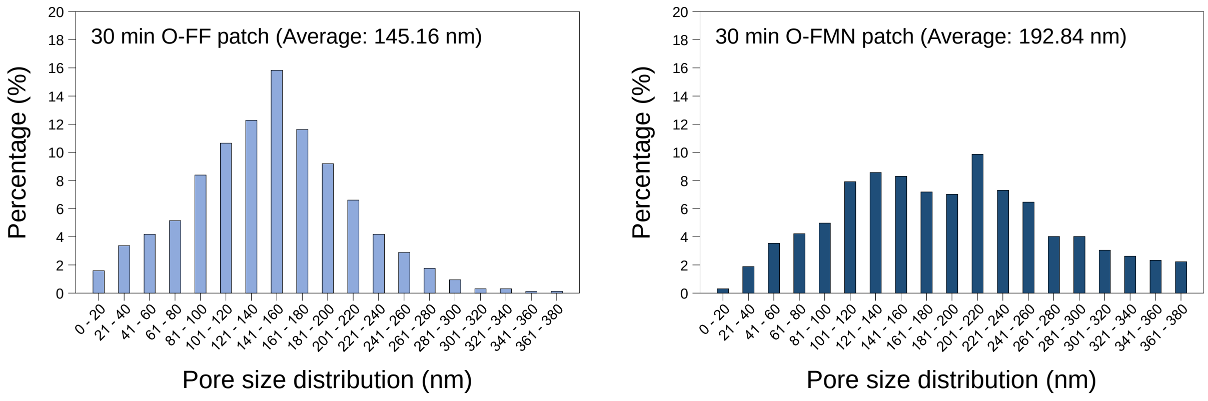

Supplementary Figure 1. Pore size distribution of 30 min O-FF and 30 min O-FMN patches. The quantification of the pore sizes (diameter) was measured using ImageJ software.

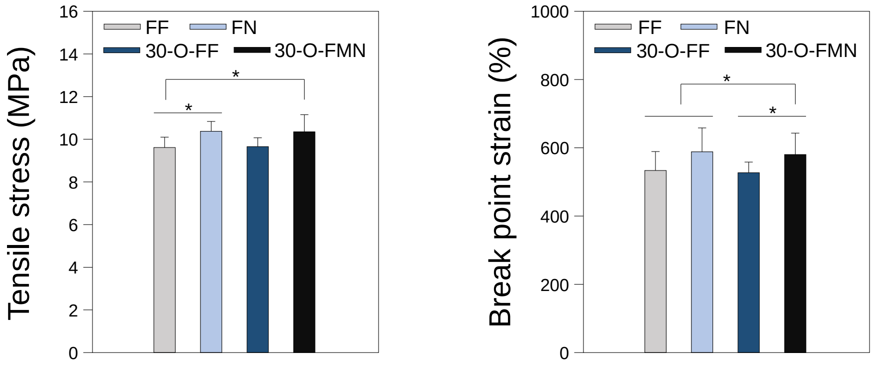

Supplementary Figure 2. Tensile stress and break point strain of the FF, FN, 30 min O-FF, and 30 min O-FMN patches (*n* = 9 for each group). Error bars = mean ± standard deviation. (*p < 0.05)

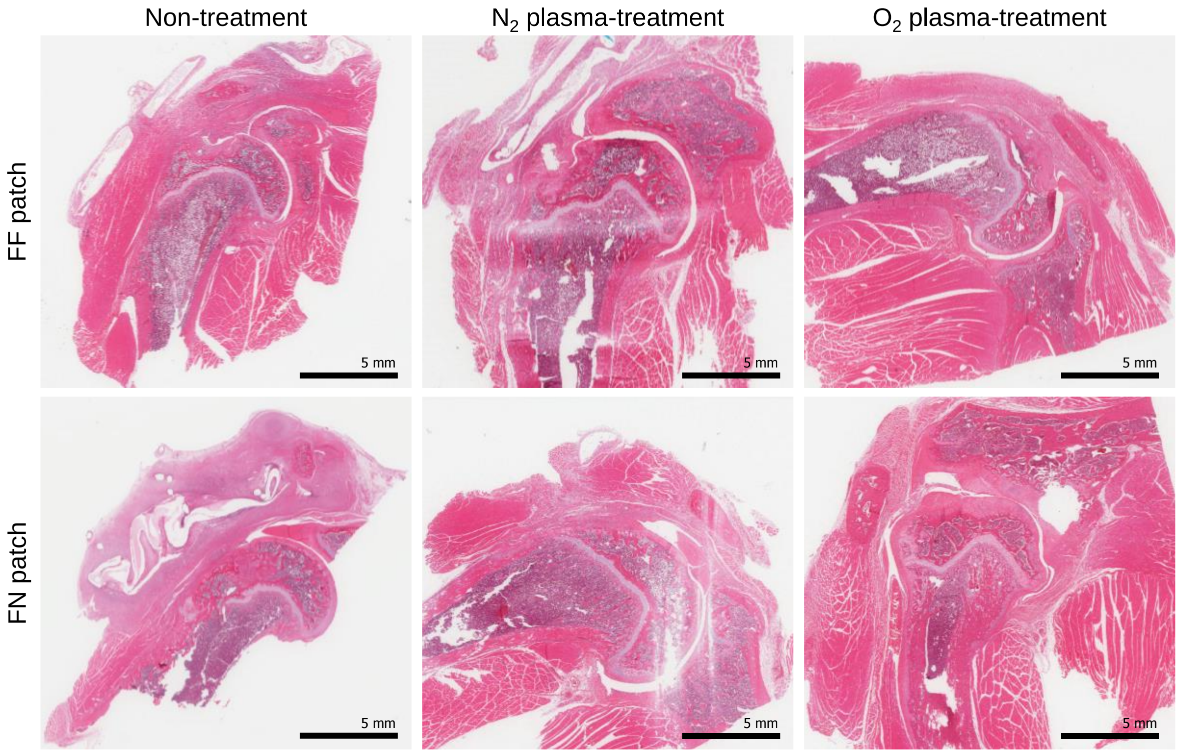

Supplementary Figure 3. Representative histologic gross images of H&E staining of the insertion site of FF, FN, N-FF, N-FN, O-FF, and O-FMN patches onto the supraspinatus tendon 4 weeks after repair.

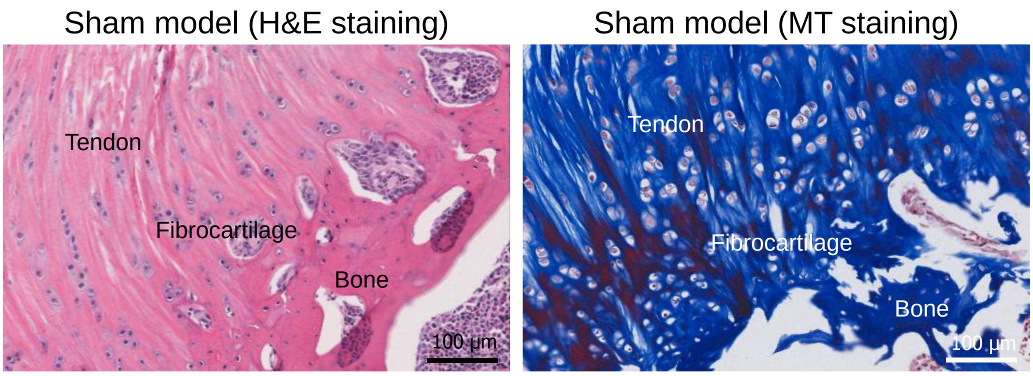

Supplementary Figure 4. Representative histologic images of H&E staining and Masson trichrome staining of supraspinatus tendon tissue of sham model.

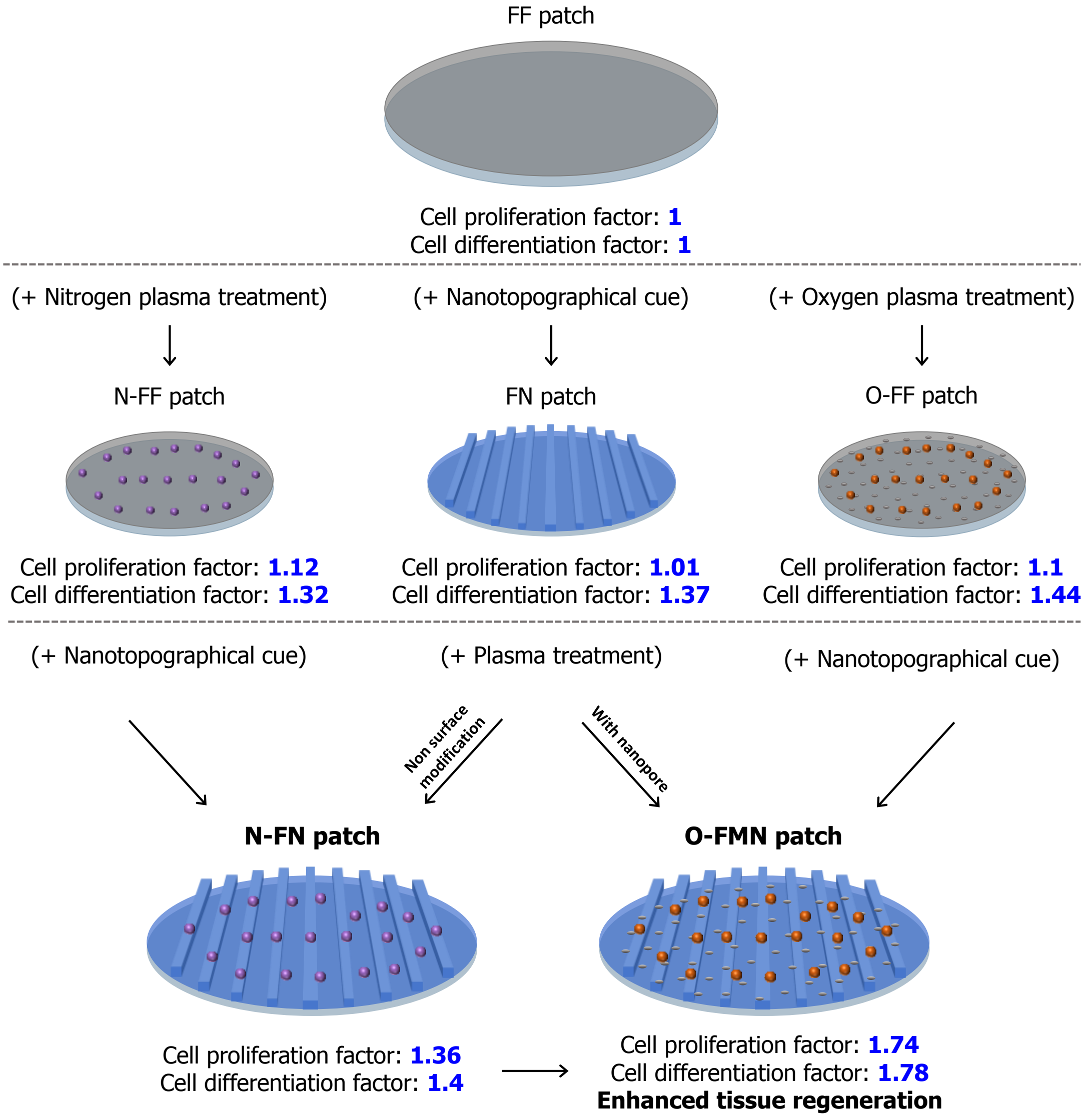

Supplementary Figure 5. Schematic model of the enhanced effects of aligned nano-topographic cues and O_2 plasma treatment on the proliferation and osteogenic mineralization of tenocytes. The relative proliferation factors and osteogenic mineralization factors were extracted from quantitative analysis of cell proliferation after 5-days culture and quantification of the osteogenesis degree with Alizarin Red staining, respectively.
