# Supplementary material for: Plasma-assisted multiscale topographic scaffolds for soft and hard tissue regeneration
Source: NPJ Regen Med. 2021 Sep 9;6:52. doi: 10.1038/s41536-021-00162-y (PMC8429553; doi:10.1038/s41536-021-00162-y)
Supplement: Supplementary file 2 — Reporting Summary [file 41536_2021_162_MOESM2_ESM.pdf]

nature research

Corresponding author(s): Jangho Kim

Last updated by author(s): May 27, 2021

Reporting Summary

Nature Research wishes to improve the reproducibility of the work that we publish. This form provides structure for consistency and transparency in reporting. For further information on Nature Research policies, see our Editorial Policies and the Editorial Policy Checklist.

Statistics

For all statistical analyses, confirm that the following items are present in the figure legend, table legend, main text, or Methods section.

n/a	Confirmed	
☐	☒	The exact sample size (*n*) for each experimental group/condition, given as a discrete number and unit of measurement
☐	☒	A statement on whether measurements were taken from distinct samples or whether the same sample was measured repeatedly
☐	☒	The statistical test(s) used AND whether they are one- or two-sided<br>*Only common tests should be described solely by name; describe more complex techniques in the Methods section.*
☒	☐	A description of all covariates tested
☐	☒	A description of any assumptions or corrections, such as tests of normality and adjustment for multiple comparisons
☐	☒	A full description of the statistical parameters including central tendency (e.g. means) or other basic estimates (e.g. regression coefficient) AND variation (e.g. standard deviation) or associated estimates of uncertainty (e.g. confidence intervals)
☐	☒	For null hypothesis testing, the test statistic (e.g. *F*, *t*, *r*) with confidence intervals, effect sizes, degrees of freedom and *P* value noted<br>*Give P values as exact values whenever suitable.*
☒	☐	For Bayesian analysis, information on the choice of priors and Markov chain Monte Carlo settings
☒	☐	For hierarchical and complex designs, identification of the appropriate level for tests and full reporting of outcomes
☒	☐	Estimates of effect sizes (e.g. Cohen's *d*, Pearson's *r*), indicating how they were calculated

Our web collection on statistics for biologists contains articles on many of the points above.

Software and code

Policy information about availability of computer code

Data collection	ToupTek ToupView (version x64, 3.7.6701) used to acquire the calcium deposition images.
Data analysis	ImageJ (1.53e) was used to analyze the images of scanning electron microscope (SEM) and contact angle. Aperio ImageScope (version 12.4.0.5043) MIMICS 14.0 3D imaging software and were used to analyze the images of the histological observation. IBM SPSS Statistics Editor (version 1.0.0.1447) was used to analyze the statistics. Microplate Manager 6 Software (version 6.0) used to analyze the absorbance. MSAT-Lite (version 1.1.1) was used to analyze the mechanical properties. Avantage (Thermofisher) was used to fit the peak of XPS data.

For manuscripts utilizing custom algorithms or software that are central to the research but not yet described in published literature, software must be made available to editors and reviewers. We strongly encourage code deposition in a community repository (e.g. GitHub). See the Nature Research guidelines for submitting code & software for further information.

Data

Policy information about availability of data

All manuscripts must include a data availability statement. This statement should provide the following information, where applicable:

- Accession codes, unique identifiers, or web links for publicly available datasets
- A list of figures that have associated raw data
- A description of any restrictions on data availability

The data that support the findings of this study are available from the corresponding author upon reasonable request.

Field-specific reporting

Please select the one below that is the best fit for your research. If you are not sure, read the appropriate sections before making your selection.

☒ Life sciences ☐ Behavioural & social sciences ☐ Ecological, evolutionary & environmental sciences

For a reference copy of the document with all sections, see nature.com/documents/nr-reporting-summary-flat.pdf

Life sciences study design

All studies must disclose on these points even when the disclosure is negative.

Sample size	Sample sizes were not predetermined based on statistical methods, but were chosen according to the standards of the field (at least three independent biological replicates for each condition), which generated a sufficient number of single-molecule trajectories and gave sufficient statistics for the effect sizes of interest.
Data exclusions	No data was excluded from this analysis
Replication	All attempts at replication were successful.
Randomization	All samples were randomized before the experiments.
Blinding	Investigators were not blinded in vitro study. Blinding during collection was not needed because conditions were well controlled. Blinding during analysis was not feasible as the differences between samples under different conditions were visually apparent in the trajectories. Blinding is also not necessary because the results are quantitative and did not require subjective judgment or interpretation. Blinding is not typically used in the field. To analysis the histological images, investigators were blinded for objective judgment.

Reporting for specific materials, systems and methods

We require information from authors about some types of materials, experimental systems and methods used in many studies. Here, indicate whether each material, system or method listed is relevant to your study. If you are not sure if a list item applies to your research, read the appropriate section before selecting a response.

Materials & experimental systems

n/a	Involved in the study	
☒	☐	Antibodies
☒	☐	Eukaryotic cell lines
☒	☐	Palaeontology and archaeology
☐	☒	Animals and other organisms
☒	☐	Human research participants
☒	☐	Clinical data
☒	☐	Dual use research of concern

Methods

n/a	Involved in the study	
☒	☐	ChIP-seq
☒	☐	Flow cytometry
☒	☐	MRI-based neuroimaging

Animals and other organisms

Policy information about studies involving animals; ARRIVE guidelines recommended for reporting animal research

Laboratory animals	Sprague-Dawley rats weighing 350–450 g and 6-week-old, male mice (C57Bl/6N) were used for the tendon and bone tissue harvest.
Wild animals	The study did not involve wild animals.
Field-collected samples	The study did not involve samples collected from the field.
Ethics oversight	The animal study was approved by the Ethics Committee of Chonnam National University Medical School and Chonnam National University Hospital (CNUHIACUC-18013).

Note that full information on the approval of the study protocol must also be provided in the manuscript.
